# 4112 Tools for Self-Management of Obstetric Fistula in Low and Middle-income Countries: A Qualitative Study Exploring Pre-implementation Barriers and Facilitators among Global Stakeholders

**DOI:** 10.1017/cts.2020.440

**Published:** 2020-07-29

**Authors:** Nessa E Ryan, Bernadette Boden-Albala, Gabriel Ganyaglo, Alison El Ayadi

**Affiliations:** 1 NYU - H+H Clinical and Translational Science Institute; 2 University of California, Irvine; 3 Korle Bu Teaching Hospital

## Abstract

OBJECTIVES/GOALS: Insertable devices (IDs) for obstetric fistula (OF) management are feasible, acceptable, but not accessible; implementation determinants in low and middle-income countries (LMICs) are unknown. Thus, the purpose of this study was to understand pre-adoption facilitators and barriers among global stakeholders for a therapeutic ID for OF in LMICs. METHODS/STUDY POPULATION: Stakeholders, including researchers (n = 11), clinicians (n = 4), government officials (n = 2), and administrators (n = 4), were purposefully identified from various sectors involved in understanding and addressing the needs of women with OF: clinical care, academia, international health, civil society, and government. Twenty-one individuals were interviewed about their perceptions of IDs for OF self-management and their implementation. Interviews were audio-recorded and transcribed. The Consolidated Framework for Implementation Research (CFIR) guided data collection and analysis. Thematic analyses were carried out within Nvivo v.12. RESULTS/ANTICIPATED RESULTS: Determinants of implementation of an ID for OF self-management (by CFIR domain) include: (1) intervention characteristics—*relative advantage* and *cost*; (2) individual characteristics—*knowledge and beliefs about the innovation*; (3) inner setting-- *organizational culture, implementation climate, tension for change*, and *compatibility*; (4) outer setting-- *patient needs and resources* and *external policy and incentives*; (5) process—*opinion leaders* and *collaboration*. Facilitators include: tension for change for low-cost, accessible IDs; relative advantage over existing tools; development of partnerships; and identification of implementation champions. Barriers include: need for educational strategies to encourage clinical provider acceptability; lack of evidence of the optimal beneficiary. DISCUSSION/SIGNIFICANCE OF IMPACT: Tools for therapeutic OF self-management could be integrated into comprehensive OF programming. Employing the CFIR as an overarching typology allows for comparison across contexts and settings where OF care occurs and may be useful for clinicians, researchers, and policy-makers interested in implementing IDs for OF self-management in LMICs. CONFLICT OF INTEREST DESCRIPTION: I am working with colleagues at the non-profit Restore Health on developing an insertable cup for therapeutic self-management of obstetric fistula in LMICs

